# Exploring the role and lived experiences of people with disabilities working in the agricultural sector in northern Nigeria

**DOI:** 10.4102/ajod.v11i0.897

**Published:** 2022-08-16

**Authors:** Precious N. Sango, Mohammed Bello, Roy Deveau, Kevin Gager, Belinda Boateng, Hauwa K. Ahmed, Mohammed N. Azam

**Affiliations:** 1 International School of Disability Studies, Jos and Abuja, Nigeria; 2 Faculty of Health and Social Sciences, School of Applied Social Sciences, University of Bedfordshire, Luton, United Kingdom; 3 African Centre for Innovative Research and Development (AFRI-CIRD), Kano, Nigeria; 4 Tizard Centre, University of Kent, Canterbury, United Kingdom; 5 Propcom Mai-karfi Programme, Abuja, Nigeria

**Keywords:** disability, agriculture, farmers, discrimination, northern Nigeria

## Abstract

**Background:**

It is estimated that over 75.0% of households in sub-Saharan Africa are involved in agriculture, and the majority of the poor in rural areas rely on agriculture for their livelihoods. One billion people living with disabilities in low- and middle-income countries are argued to make up the poorest of the poor, yet to our knowledge, no literature has captured the livelihood of people living with disabilities in the context of farming in Nigeria, specifically northern Nigeria where most of the households are involved in agriculture and related activities.

**Objectives:**

This article reports on findings from a study that sought to understand disability in the context of northern Nigerian farming, with a particular focus on the role and lived experiences of people living with disabilities working in the agricultural sector.

**Method:**

A survey questionnaire was developed and captured the experiences of 1067 people living with disabilities working in the agricultural sector across five states (Adamawa, Bauchi, Jigawa, Kaduna and Yobe) in northern Nigeria.

**Results:**

Findings indicate that people with disabilities are actively participating in agricultural activities for several reasons, which specifically included ‘forced to and for survival’. When participants reported needing care, this was predominantly provided by family members. Findings also showed that participants with disabilities experienced several economic and sociocultural challenges because of their impairments.

**Conclusion:**

This study adds to the very limited literature on farmers living with disabilities in sub-Saharan Africa and so highlights the need for more research to be conducted with farmers living with disabilities in Nigeria, particularly female farmers living with disabilities. These will provide more evidence pertaining to the experiences of farmers living with disabilities in order to provide effective disability- and gender-inclusive agricultural and entrepreneurship programmes in Nigeria.

**Contribution:**

The results of this research reveal important insights relating to the experiences of farmers living with disabilities in northern Nigeria, which can contribute to informing future developmental projects to achieve effective inclusion and actively benefit people living with disabilities.

## Introduction

According to Moyo (2016), 75% of the population in sub-Saharan Africa (SSA) is involved in farming and related employment, and the majority of the poor live in rural areas where their livelihoods derive from agriculture. This has important implications for Nigeria’s poverty alleviation plans, as it is estimated that in 2012, 69% of the poor in the country derived their livelihood from agriculture (NBS 2012). The agricultural sector is specified to be the highest employer of labour in Nigeria and significantly contributes to employment and economic growth (Oluwatoyese, Applanaidu & Razak 2016). Ogunlela and Mukhtar (2009) suggested that agricultural growth is central to development, food security and poverty alleviation and the sustainability of natural resources (Acharya 2006) in rural Africa. Ojiako, Idowu and Ogbukwa (2014) emphasised the importance of credit and loanable funds or capital as crucial to increasing the development of agriculture and rural economy and reported that most of their participants benefited from cooperative credits and credits from their social networks such as friends and relations; participants had limited access to modern improved technologies (Ogunlela & Mukhtar 2009) and were less likely to receive assistance from microfinance and commercial banks (also see Ogunlade et al. 2016). Some of the reasons for the latter are related to high demand for collateral and limited documentation, for example, farmers not registering their properties and assets, high risk and general aversion of banks towards agriculture (Abraham 2018; Ojiako et al. 2014).

The social and demographic characteristics of participants in research (e.g. Abraham 2018; Ogunlade et al. 2016; Ojiako et al. 2014) on farmers located in different parts of Nigeria seem to mostly include married male farmers without any disability, with the majority possessing primary school education and farming experience ranging from 10 years or more. The predominance of male farmers living without disabilities in agricultural research in low- and middle-income countries (LMICs) may be because of sociocultural, statutory and customary laws and economic components (Huyer 2016; Ogunlela & Mukhtar 2009; World Bank 2009). As presented in World Bank (2009), the design of policies and projects incorrectly assume that farmers and rural workers are mainly men. This is mainly because even though women are said to be the main farmers or producers in some parts of the world such as SSA, their roles are often not acknowledged, and they face more obstacles compared with their male colleagues in market access, product assets and services, land, labour, financial services, technology and other inputs (see Imonikebe 2010; Ogunlela & Mukhtar 2009; Sheahan, Barrett & Sheahan 2014). For example, crop choice has been mostly determined by gender in Nigeria, although these associations of women and men with specific crops and livestock products is diminishing to some extent in parts of the country (see Farnworth et al. 2020). Northern Nigeria is said to be where most households are involved in farming (Abraham 2018). None of the literature on agriculture in Nigeria reviewed included farmers living with disabilities, and Aranda-Jan (2021) and Ahlenbäck, Lee and Coe (2020) observed that there is very limited evidence based on the experiences of farmers living with disabilities in LMIC.

In Nigeria, approximately 25 million people live with a disability; 3.5 million of these have very significant difficulties in social and physical functioning (WHO & The World Bank 2011). According to Sango (2017), the lack of up-to-date data on the characteristics and experiences of people with disabilities in Nigeria and negative attitudes towards persons with disabilities in the country (Audu et al. 2013; Etieyibo & Omiegbe 2016) may contribute to the lack of prioritising of disability issues, delay in the identification of the needs of individuals with disabilities and the implementation of policies to improve their quality of life. People with disabilities have several vulnerabilities, which means they are at increased risk of being marginalised in societal life (Cooper et al. 2007; Emerson & Parish 2010).

Nigeria is characterised by distinct ethnic groupings (over 250 ethnic groups), varying in sociodemographic and sociocultural practices and religious beliefs (see Sango 2013). These ethnic groups speak over 500 different languages with different cultures and traditions (Central Intelligence Agency 2022). The main languages used in Nigeria are English, Hausa, Yoruba, Igbo and Pidgin English. The country is religiously diverse with more Muslims in the north and more Christians in the southern parts of the country (see Central Intelligence Agency 2022). These cultural and religious differences have disablist (Thomas 2007:73) implications related to stigma and exclusion for persons with disabilities in Nigeria (see Abang 1988; Obiakor 1990; Sango 2017). According to Sango (2017), the *Discrimination Against Persons with Disabilities (Prohibition) Act, 2018* in Nigeria proposed changes in the national provision for people living with a disability, including, free regular medical care, free education, transportation and subsidised housing for people living with disability, but these are yet to be implemented. Care for people with disabilities in Nigeria continues to be mainly provided by families, charities, indigenous and religious communities (see Sango 2017).

Evidence (e.g. Banks & Polack 2014; Groce et al. 2011; Mitra, Posarac & Vick 2013) is beginning to highlight the association between disability and poverty, indicating that 80% of the world’s 1 billion people living with disability residing in LMIC such as Nigeria are ‘poorer than their peers living without disabilities in terms of access to education, access to healthcare, social support, civic involvement, employment and income’ (WHO & World Bank 2011). Although the causal associations between disability and poverty are poorly understood (Banks & Polack 2014:i; Groce et al. 2011:1493–1495; Mitra et al. 2013:1–3), it has been suggested that disability is ‘both a cause and consequence of poverty’ and that poverty and disability ‘reinforce each other, contributing to increased vulnerability and exclusion’ (DFID 2000:1–2; Ofuani 2011; Trani & Loebe 2012:S19). By way of illustration, Groce et al. (2011) argued that disability can heighten poverty because of the institutional, environmental and attitudinal barriers people living with disabilities face in their daily lives thus contributing to their social exclusion. Concurrently, poverty can amplify the chances of having a disability as chronically poor individuals are often at risk of ill health, injuries, malnutrition, inadequate access to public health services and poor living conditions such as lack of safe water and unsafe work environments which could lead to disability. These factors are augmented by conditions of conflict and other humanitarian emergencies in many developing countries such as Nigeria (see Groce et al. 2011).

This research in a topic area with limited evidence (e.g. Ahlenbäck et al. 2020; Aranda-Jan 2021) on disability and agriculture in LMIC has the potential to highlight the importance of agribusiness in addressing barriers faced by farmers living with disabilities. These barriers are worsened by the lack of recognition relating to the crucial role agriculture plays as a potential means of livelihood for people living with disabilities (Aranda-Jan 2021), and often agricultural inclusion is said to focus on the gender gap rather than the disability gap (Aranda-Jan 2021).

### Research’s aim and questions

This article reports findings from a larger project funded by the United Kingdom’s (UK) Department for International Development (DFID), Propcom Mai-karfi and Palladium, which sought to explore ways in which people living with disabilities can be better included in agribusiness in northern Nigeria. ‘Disability’ in the context of this study is defined as persons:

[*W*]ho have long-term physical, mental, intellectual or sensory impairments which in interaction with various barriers may hinder their full and effective participation in society on an equal basis with others. (United Nations Convention on the Rights of Persons with Disabilities 2006:4)

Specifically, this article addresses the following objectives:

- Describe the characteristics, roles and economic activities (including associated work-related risks) of those living with disabilities engaged in the agricultural sector in northern Nigeria
- Explain the ownership structure, financial inclusion, access to and use of agricultural assets by participants living with disabilities
- Examine how farmers with disabilities access information, training and market opportunities in the agricultural sector
- Describe sociocultural implications such as the interplay between gender and disability within the agricultural sector.

## Methods

Quantitative data were collected, using a survey questionnaire devised for this study, from participants living in the UK DFID’s Propcom Mai-karfi-Palladium-funded project implementation states in northern Nigeria, specifically Adamawa, Bauchi, Jigawa, Kaduna and Yobe states.

### Eligibility for inclusion in the study

Participants were eligible for inclusion in this study if they met the following criteria:

- Youths and adults (16 years and over) with any type of disability residing in Adamawa, Bauchi, Jigawa, Kaduna and Yobe states, Nigeria and involved in some form of agricultural livelihood or livelihoods related to agricultural activities, for example, trading, marketing and civil servant roles related to agriculture
- Those who could communicate in the English language or one of the local languages (Hausa, Fulani or Kanuri); in cases where the respondent could not communicate in any of the languages, sign language was deployed and responses provided by the respondent were further validated with the caregiver.

### Ethical considerations

This research was given ethical approval by the Research and Ethics Committee of Bayero University, Kano (BUK), Kano State, Nigeria. All adult participants signed a consent form after being given information about the study. For participants below 18 years of age (16 and 17 years of age), consent was sought from their caregiver and guardian. Participation in the research was voluntary and participants were repeatedly informed of their right to withdraw and remove their information from the research at any time, without sanction. Participants were informed that the information provided would be completely confidential and identifiable information would not be shared.

### Sampling and procedure

Three Local Government Areas (LGAs) that are part of the UK DFID’s Propcom Mai-karfi-Palladium-funded programme in northern Nigeria were selected from each of the five states (Adamawa, Bauchi, Jigawa, Kaduna and Yobe states). This was an exploratory study where scarce data were available on those farmer living with disabilities, as such a multistage sampling procedure was used to select the study sample within the selected states. Firstly, disabled persons’ organisations (DPOs), social welfare departments of LGAs, district heads (DHs), ward heads (WHs) and religious groups (RGs) were approached to identify individuals with disabilities in their communities. Secondly, a random sample was taken of participants referred by DPOs. Finally, all referred potential participants completed the Washington Group Extended Set on Functioning (WG-ES) questionnaire and only individuals who had functional difficulties based on the WG-ES questionnaire were surveyed. The study aimed for a total of 12 000 samples (1067 was achieved), with 200 sample sizes for 3 states (Bauchi, Jigawa and Yobe), while Kaduna and Adamawa had the highest target of 300 each because of their population number compared with the other states. The targeted sample size was based on inputs from the DPOs, stating an average size of 600 people living with disabilities can be found in each of the locations where the UK DFID’s Propcom Mai-karfi-Palladium was working. Accessibility to people living with disabilities across the locations of UK DFID’s Propcom Mai-karfi-Palladium intervention was a major constraint to obtaining the target sample size.

The survey questionnaire was administered by 25 research assistants (RAs) who recorded responses via KoBoToolbox (Toolbox 2016), an open-source suite of tools for digital data collection. The 25 RAs (5 per state) were under the supervision of a state supervisor from the African Centre for Innovative Research and Development (AFRI-CIRD). Research assistants were trained in research ethics and in standardised administration of the survey and use of KoBoToolbox. A 1-day pilot study of the survey questionnaire was conducted to confirm the validity of survey questions and results were computed. Survey interviews conducted were administered and recorded in either English, Hausa, Fulani or Kanuri; these lasted around 2 h each and were conducted between the months of July and August 2019. The interviews were transcribed and translated to the English language by the same RAs who administered the survey; this helped to maintain the validity and integrity of data collected. Using RAs to administer the questionnaire supported participation; however, it may also introduce various systematic biases through asking RAs to interpret and enter data during the interviews. Steps were taken through training and data validation to avoid such systematic biases. While this sample was of a practical size, it does reflect the experiences of specific geopolitical areas of Nigeria and may not be generalisable to all parts of Nigeria or neighbouring African countries. The research sample did not adequately capture the perceptions and experiences of mental health and intellectual disabilities. Why they were not adequately represented is not clear, but it could possibly depend on how the questions were translated and the perceptions, understanding and attitudes of respondents or the caregivers who supported participants.

### The survey questionnaire

The survey questionnaire (Understanding Disability in the Context of northern Nigeria) was designed jointly by the International School of Disability Studies (ISDS), Propcom Mai-karfi and AFRI-CIRD. The questionnaire was based upon reading relevant disability literature and examples of similarly focused surveys conducted in an African country (e.g. Montrose 2017). The questionnaire had the following sections:

- *Profile of respondents*: Participant characteristics, for example, age, gender, disability type, educational attainment, type of agricultural activity, living and support arrangements.
- *Roles, economic activities and work-related risks*: Agricultural enterprise, for example, rice farming, cattle-rearing. Specific agricultural activities, for example, ploughing, planting and weeding. Membership of agricultural cooperative and responsibility or leadership role played.
- *Ownership, access and use of agricultural assets*: Assets used in employment, for example, land, motorbikes, tractors, hoes, safety boots, whether the assets are owned, rented or borrowed; ability to use assets unaided or requiring hired labour; presence of cultural norms, which impeded personal use of the assets.
- *Decision making on agricultural enterprise*: Why and who decided to engage in current agricultural activities, paying for required goods and services? Who made decisions on what to do with harvest and financial proceeds?
- *Access to market information and capacity-building opportunities related to questions within the survey*: Does the person’s disability enhance or limit their ability to receive training, access buyers and access market prices related to agricultural activities?
- *Awareness of laws on disability and influence of social norms and rights on agricultural activities*: Participant’s experiences or knowledge of social norms and formal or informal laws around disability, whether these are an advantage or disadvantage regarding agricultural activities and whether these can be managed.
- *Interfacing with NGOs or INGOs*: Receipt of assistance from NGOs and which ones.
- *Gender and disability in agricultural activities*: Participants’ views regarding equal treatment of women and adolescent girls in agribusiness; female involvement in agriculture, for example, soybean, maize farming; receipt of support to carry out agribusiness; organisations that support ‘economic empowerment’ of women with disabilities; support received; employment in community organisations; types of agriculture suited to women; equality of support provided with men.
- *Financial inclusion and access to finance*: Ownership of bank accounts and type of accounts; access to digital or mobile banking; organisations providing financial assistance for agricultural activities; receipt of both formal and informal loans and if not, how agricultural activities are funded; reasons for not receiving loans.
- *Personality traits and attributes*: Participants asked to suggest their individual personality traits from a provided list and whether these supported or limited their agricultural activities (e.g. honest, trustworthy), reported elsewhere.
- *Poverty Probability Index* (*PPI; Grameen Foundation 2014*): The PPI is a tool used for estimating the probability or likelihood of a household falling below a certain poverty line. The PPIs are country-specific and include a set of 10 standardised questions (Grameen Foundation 2014) that are most strongly correlated with poverty, as determined from the country’s household income and expenditure survey.
- *Household Dietary Diversity Score* (*HDDS; Swindale & Bilinsky 2006*): The HDDS examines the diversity of diet for households; a more diverse diet indicates a greater range of food types being consumed. A more diverse diet is highly correlated with caloric and protein adequacy, percentage of protein from animal sources (high-quality protein) and household income.

Response categories included closed and open-ended questions, with many questions using both close- and open-ended responses. Several questions asked participants to respond to a list with *yes* or *no* (and included an ‘any other category’) and an ‘explain your response’ question. For example, the survey question number 2.7 (‘Does your disability prevent you from taking on any roles or responsibilities within your organisation?’) was responded to initially with ‘yes’ or ‘no’, followed by, ‘If yes, what type of disability allows this restriction?’ ‘1 = difficulty seeing’; ‘2 = difficulty hearing’; ‘3 = difficulty remembering or concentrating’; ‘4 = difficulty with self-care’; ‘5 = difficulty communicating or being understood’; ‘6 = difficulty climbing stairs’; followed by, ‘explain your response’. Some open-ended questions were also employed, for example, question number 6.1: Are there any social norms or formal or informal laws around disability that you have that puts you at an advantage when it comes to managing your agricultural activities? Explain your answer.

Most questions allowed for additional comments and included questions related to the effect of the person’s disability upon the responses provided. For example, regarding leadership roles in agricultural cooperatives, participants were asked, ‘Does your disability limit your capacity in a leadership role? Explain your answer’. Only the quantitative findings from the measure are reported in this article.

### Data analysis

Data from the quantitative survey were analysed using SPSS 25 (Morgan et al. 2019). Results are presented using descriptive statistics and statistical significance was tested using cross-tabulation.

## Results

### General characteristics of participants

Table 1 shows the distribution of participants by gender, age, state of residence and type of disability and living circumstances by gender and support provision.

**TABLE 1:** General characteristic of participants.

Characteristic	Male		Female		Total	
	*n*	%	*n*	%	*n*	%
Gender	754	70.7	313	29.3	1067	100
Age (years)						
15 and under	1	0.1	4	0.4	5	0.5
16–25	132	17.5	74	23.6	206	19.3
26–35	193	25.6	76	24.3	269	25.2
36–45	175	23.2	69	22.0	244	22.9
46–55	122	16.2	44	14.0	166	15.6
56–65	80	10.6	19	6.1	99	9.3
66 and above	51	6.8	27	8.6	78	7.3
Totals	754	100	313	100	1067	100
**State of residence**						
Adamawa	151	20.0	66	21.1	217	20.3
Bauchi	166	22.0	41	13.1	207	19.4
Jigwa	136	18.0	69	22.0	205	19.2
Kaduna	165	21.9	74	23.6	239	22.4
Yobe	136	18.0	63	20.1	199	17.8
Totals	754	100	313	100	1067	100
**Educational attainment†**						
Islamic education‡	271	35.9	126	40.3	397	37.0
Primary	81	10.7	32	10.2	113	11.0
Secondary	202	26.8	56	17.9	258	24.0
Tertiary	89	11.8	23	7.3	112	10.0
No formal education	97	12.9	72	23.0	169	16.0
Other	14	1.9	4	1.3	18	2.0
Total	754	100	313	100	1067	100
**Main Disability§**						
Difficulty climbing stairs	347	46.0	146	46.6	493	46.2
Difficulty seeing	203	27.0	90	28.8	293	27.5
Difficulty hearing	173	22.0	58	18.5	231	21.6
Difficulty with self-care	23	3.0	13	4.1	36	3.4
Difficulty remembering or concentrating	8	1.0	3	1.0	11	1.0
Difficulty communicating	0	0.0	3	1	3	0.3
Total	754	100	313	100	1067	100
**Support**						
Living at home with support from unpaid carers (e.g. partner, family, friends).	524	69.5	255	81.5	779	73.0
Living at home, no support required.	174	23.1	39	12.5	213	20.0
Living at home with support from paid carers.	50	6.6	15	4.8	65	6.1
Living in a long-stay hospital or community care or sheltered housing.	6	0.7	3	1.0	9	0.8
Other	0	0.0	1	0.3	1	0.1
Total	754	100	313	100	1067	100¶

† , Educational attainment is the highest level achieved by the participant;

‡ , Through Islamic education platforms, people also acquire business and life skills built on the principles of cooperation and self-reliance. They are provided with job-acquiring skills, including leadership skills that facilitate mobility towards self-reliance. Entrepreneurship skills are also provided through Islamic schools, especially on household-level businesses for women. This includes soap and handwash-making, bead production, knitting, etc.;

§ , Main disability derived from Washington Group Extended Set on Functioning (WG-EF);

¶ , Rounding factors lead to some columns not adding to 100%.

The main disabilities reported by the participants were physical, visual and auditory. Participants gave the cause of their disabilities as: 23.1% ‘accidents’; 21.4% ‘born with it’; 4.9% ‘old age’; 4.3% caused by ‘religious’ or ‘traditional beliefs’ and 3.4% ‘conflict in my community’. The remainder, just over 40.0% of participants, reported ‘other’ factors as causes of disability; when prompted, illness because of conditions such as polio, glaucoma, leprosy, snake bite infections, childhood illnesses, meningitis or fever were mentioned. The highest level of education attained by the 1067 participants is shown in Table 1. The difference in the highest educational attainment level between males and females was significant (chi-square 27.383 [*df* = 5], [*p* < 0.000]). Women were more likely to report having no formal education and lower levels of secondary and tertiary education.

Table 1 also displays the living and support arrangements experienced by the participants. A greater proportion of male participants lived at home without support than female participants because it was not required, as most frequently they lived at home supported by unpaid carers (i.e. family members). The PPI measure of poverty showed that this sample experienced very high levels of poverty. A total of 69.0% of participants had a high probability of living on less than US$2.00 per day. The diet of the participants was biased towards cheaper foods. The average HDDS score of 6.3 suggested that only 11.3% of the households comprising of participants living with disabilities in northern Nigeria consumed a reasonable mix of all food groups. These households most commonly ate meals containing cereals (86.9%, *n* = 927) and vegetables (64.9%, *n* = 692), roots and tubers (48.2%, *n* = 516) and meat (35.9%, *n* = 383).

### Employment

Participants reported having their main employment in two main types of agriculture: crop farming and livestock farming (see Table 2). Many participants undertook elements of each main type of agriculture and other agricultural employment, in addition to their main agricultural employment. For example, a (main) crop farmer may also keep chickens or other livestock and work as an animal welfare worker or civil servant. In all, 42.0% of participants had no employment other than agriculture. A range of different crops and livestock are included in the two main categories, for example, crop farmers included growing rice, beans, millet or maize and livestock included rearing chickens, cattle and pigeons.

**TABLE 2:** All types of agricultural activity† by gender.

Agricultural activity	Male (%)	Female (%)
Crop farming	84	63
Livestock rearing	57	53
Agro-processing	5	7
Animal health worker	0	1
Input dealer	1	0
Others	2	6

† , Participants were often engaged in more than one agricultural activity.

As seen in Table 2, other forms of agriculture practiced to a much lesser degree included agro-processing, animal health workers, input dealers and others. A total of 42.0% of participants engaged solely in agricultural work, while 23.2% of participants also engaged in other, non-agricultural employment. Other forms of employment include ‘unspecified’ (20.4%), civil servants (4.9%) and artisans (9.1%). A pattern was observed that those who also worked in non-agricultural sector reported higher levels of educational attainment, for example, participants working as artisans (36%) reported having secondary school education as their highest educational attainment while those reporting as civil servants (44%) reported tertiary level as their highest educational attainment.

The main reasons given for working in agriculture were the following: ‘forced to do for survival’ *n* = 526 (49.3%), ‘passion or interest’ *n* = 404 (37.9%), ‘family influence’ *n* = 125 (11.7%). When asked who made the decision to work in agriculture, 85.0% reported they made that decision for themselves and 15.0% reported that others influenced them. Caregivers and family members were reported to provide most of the influence rather than other community members.

Disability played a role in restricting the agricultural activities that participants were able to undertake without support, that is, having to pay for hired help or rely upon unpaid help from family members. Three-quarters of the sample said that their disability restricted the agricultural activities they could undertake without assistance from other people. Participants with physical, visual and hearing disabilities reported being most in need of assistance with agricultural tasks, for example, ploughing, transportation of farm products to storage, harvesting, weeding and planting; 45.6% (*n* = 368) of those with difficulty climbing stairs needed assistance, while 31.4% (*n* = 253) with visual difficulties and 16.5% (*n* = 133) with hearing disability needed help as well.

Participants reported using various forms of assistance to carry out their work and other daily activities. The main forms of assistance used reflected the participants’ particular experience of disability and included the use of walking aids or sticks (30%), wheelchairs (27%), hearing aids (14%), sign language training (6%) and record-keeping training (4%).

### Family economic contribution

People living with disabilities in northern Nigeria contribute significantly to the household incomes, with just under one-half being the main breadwinners. Table 3 shows a greater proportion of men than women being the main breadwinners.

**TABLE 3:** Household financial contribution by gender.

Household financial contribution	Male		Female	
	*n*	%	*n*	%
Main breadwinner	459	60.9	33	10.5
Contribute to family income	249	33.0	214	68.4
No financial contribution	46	6.1	66	21.1

**Total**	**754**	**100**	**313**	**100**

### Leadership roles in the community

As illustrated in Table 4, while this sample of people living with disabilities shows that some have leadership roles in their communities, they do not seem well-represented at this level. A greater proportion of men with disabilities hold community leadership roles than women with disabilities, especially in organisational leadership roles.

**TABLE 4:** Leadership roles in the community.

Roles in the community	Female		Male		Total	
	*n*	%	*n*	%	*n*	%
None	208	66.5	353	46.8	561	52.6
Kinship or head of family roles	43	13.7	208	27.6	251	23.5
Religious leadership roles	51	16.3	132	17.5	183	17.2
Organisational leadership roles	9	2.9	151	20.0	160	15.0
Traditional leadership roles	9	2.9	37	4.9	46	4.3
Community development roles	2	6.0	23	3.1	25	2.3
Other	4	1.3	8	1.1	12	1.1
Elected position roles	1	0.3	4	0.5	5	0.5
Market leadership roles	1	0.3	4	0.5	5	0.5

**Total**	**313**	**100**	**754**	**100**	**1067**	**100**

Agricultural cooperatives are one community organisation in agricultural-based societies; 92% of participants did not belong to any agricultural cooperative. Of the 8% that did, these included: ‘Cripple poultry farming cooperative society’, ‘Rice Farmers Association of Nigeria’ and ‘Bauchi state deaf farmers’ cooperative society’. When asked why they did not belong to an agricultural cooperative, half of the participants attributed this to their disability.

### Assets: owned, rented or borrowed

Table 5 shows the level of assets available for use by participants to undertake farming activities and the percentage of those who are owned, rented or borrowed. The high levels of basic equipment (e.g. hoes, cutlasses and water troughs) available for use contrast with the low availability of more expensive mechanised assets such as tractors and motorbikes.

**TABLE 5:** Assets: Available for use, owned and rented or borrowed.

Asset	Available for use		Owned % of available for use	Rented or borrowed % of available for use
	*n*	%		
Hoe	876	82.1	86	14
Cutlass	811	76.0	83	17
Land	617	57.8	56	44
Animal traction	192	18.0	18	82
Tractor	44	4.1	2	98
Motorbike	61	5.7	41	57
Knapsack sprayer	134	12.6	43	58
Harvester	64	6.0	55	44
Water pump	81	7.6	38	61
Wheelbarrow	117	11.0	60	40
Safety boots	58	5.4	97	3
Nose mask	45	4.2	89	11
Animal pen or house	164	15.4	93	7
Watering trough	155	14.5	94	6
Feeding trough	215	20.1	96	3
Storeroom	188	17.6	86	13
Others	25	2.3	56	16

There were no significant differences by gender for assets available for use apart from: land (males 58%; females 49%, chi-square 31.155 (*df*1), *p* < 0.000), motor bike (males 43%; females 25%, chi-square 8.211 (*df*1), *p <* 0.004) and knapsack sprayer (males 43%, females 39%, chi-square 10.950 [*df*1], *p* < 0.001).

### Access to financial and other capacity-building services and financial decision-making

Seeking and using financial advice, information and services may be important in agricultural enterprise. For example, financial services are particularly important for increasing agricultural development and economy. A majority (68%) did not have a bank account, with a significantly greater proportion of women (80%) than men (64%) not having access to a bank account (chi-square 24.992 [*df*1] *p* < 0.000). The most common reason for not having a bank account, reported by 90% of the participants, was having no money to save.

Most participants lacked awareness of organisations that provide financial assistance to support agricultural activities in their communities (such as the microfinance institutions [MFIs], Bank of Agriculture [BOA] and Anchor Borrowers Scheme by the Central Bank of Nigeria, which specifically targets rural smallholder farmers). A significantly greater proportion of men (41%) had no awareness of such organisations than women (32%) (chi-square 7.25 [*df*1] *p* < 0.01). A total of 38% of participants reported having no such awareness. Access to funds for agricultural activities was predominantly through borrowing from friends (*n* = 443) or self-financed (*n* = 868).

### Access to opportunities for capacity-building and business information

There are a couple of opportunities for farmers to develop and improve their farming techniques and business skills in Nigeria. These include those provided by the Small and Medium Scale Development Agency of Nigeria (SMEDAN), BOA, Nigeria Incentive-Based Risk Sharing System for Agriculture (NIRSAL) and others. The following four factors determined whether participants’ disability limited or enhanced their ability to access four different services: (1) training in agricultural techniques, (2) goods and services required to support their work, for example, seeds, pesticides and fertilisers, (3) access to buyers of products and (4) access to market prices for products to achieve the best price available (as seen in Table 6).

**TABLE 6:** The influence of disability upon access to services.†

Service	Disability limited access (%)	Disability enhanced access (%)
Training in agriculture	79	21
Goods and services	82	18
Access to buyers of produce	81	19
Access to market prices	81	19

† , There were no significant differences between the different disabilities and levels of limitation or enhancement to accessing services.

### Disability: Decision-making and assistance needed to undertake agricultural tasks

The majority of participants (85.4%, *n* = 911) reported that they decided on the agricultural activities undertaken. Of the *n* = 156 participants who consulted others, caregivers (42.9%, *n* = 67) and family members (40.4%, *n* = 63) provided most of the help, with spouses (6.4%, *n* = 10) mentioned the least. Similarly, 83.0% (*n* = 886) of participants reported having sufficient funds to access the goods and services they felt were required to engage in their level of agriculture (e.g. fertilisers).

Deciding what happens to agricultural produce harvested and the income received are important indicators of autonomy and economic empowerment. The individual responses were restricted to farming alone and not household or farmland jointly owned. In this sample, 35% reported making such decisions on their own, 30% said family members were involved and 17% stated that spouses or caregivers were involved. Although participants generally make most of the decisions on what to do with produce after harvest, significant differences exist by state (*x*^2^ = 0.000), gender (*x*^2^ = 0.000) and education (*x*^2^ = 0.000). Family members appear to make more of the decisions on how to manage the outcome of the harvest than participants in Adamawa (48%) and Bauchi (47%), while caregivers play the most important role in the decision-making process in Yobe (50%) than in any other states. Spouses were also reported to make such decisions in Adamawa (28%) more than in Kaduna (8%). Moreover, male participants (39%) were found to have more authority over the decision-making process than female participants (25%). Female participants reported relying more on their family members (33%), spouses (19%) and caregivers (22%) than male participants.

Furthermore, 76% of the participants reported that their disability restricted the agricultural activities they could undertake, and Table 7 shows the agricultural activities for which participants used additional help and from whom they received this help.

**TABLE 7:** Disability: Decision-making and assistance needed to undertake agricultural tasks.

Activity	Hired labour (%)	Family labour (%)	Others (%)
Ploughing	64	26	9
Transporting to storage	59	34	7
Harvesting	54	40	6
Weeding	50	42	7
Planting	46	47	7
Selling	31	61	8
Herding	43	44	12
Marketing	33	59	8
Other	46	34	20

### Social norms and rules: Both informal and formal legislation and policy

Nigeria and individual states have enacted laws to protect the rights and freedom of persons with disabilities and protect them from discrimination. Such legislation and policies are only useful if they have been implemented and people are aware of their existence. Awareness of such laws, for example, at the national level (the Nigerian *Discrimination Against Persons with Disabilities [Prohibition] Act, 2018*) and at a state level such as the *Bauchi State Disability Bill Law* in this sample was low; 86.0% said they were not aware of any national or local laws. The presence of informal social norms may have an even more powerful influence on the lives of people living with disabilities. Only 4.0% described discriminatory social norms in the context of managing agricultural activities, and when asked, ‘are there any other known social norms or informal rules in your community that prevent you from accessing the resources you need for your agricultural activities?’, 12 (1.1%) participants said yes. However, when asked about wider discrimination in their communities, participants reported that they faced discrimination of varying types and in various places in their communities, including places of worship, markets and work environments. Participants felt that people without disabilities assumed degrading stereotypes when engaging with them, because they were accustomed to seeing people living with disabilities begging or being idle on the street in their communities.

## Discussion

This article reports the findings of a survey questionnaire that sought to understand the role and experiences of people living with disabilities engaged in agricultural activities in northern Nigeria. The findings suggest that both male and female participants in the study experienced various barriers related to economic outcomes and sociocultural experiences.

Social and demographic characteristics of our participants differed in some parts from the reviewed literature on farmers living without any disability (e.g. Ogunlela & Mukhtar 2009; Ojiako et al. 2014), for example, our participants reported religious, specifically Islamic, education as their highest educational qualification, followed by no formal education. Most participants reported living with family at home with support from unpaid caregivers (i.e. partners, family and friends). This reinforces previous literature suggesting that despite the *Discrimination Against Persons with Disabilities (Prohibition) Act, 2018*, social care provision in Nigeria remains largely unchanged and mostly provided by families (see Sango 2017), with no funding from the government. Several studies (e.g. Malli et al. 2018; Murphy et al. 2007) have illustrated how limited funding and unpaid caregiver’s (family) responsibilities can negatively impact the well-being and careers of people living with disabilities.

Similar to Ojiako et al. (2014); Ogunlela and Mukhtar (2009) there were more male than female participants in our study, even though some evidence (e.g. Imonikebe 2010; World Bank 2009) suggests that women do make up most farmers in LMIC but that women remain invisible because they face more barriers than men in most aspects of agriculture. Our findings further corroborate literature reviewed (e.g. Farnworth et al. 2020) involving farmers living without disabilities in the context of gendered crop choices, where more male participants were involved in crop farming, livestock rearing and input dealing than women, who were more involved in agro-processing and animal health work than male participants.

It could be argued that our findings also shed light on issues of intersectional barriers faced by female farmers living with disabilities. These seem to be highlighted by more male participants than female participants in our study reporting being breadwinners and holding family and community leadership roles. Likewise, more male participants attended primary, secondary and tertiary formal education than female participants, who were more likely to have undertaken Islamic education or received no formal education. Other obstacles relate to what has been termed ‘customary’ discriminatory practices against women, for example, ownership of assets, the ownership of land and of bank accounts (see Imonikebe 2010; Ogunlela & Mukhtar 2009; Sheahan et al. 2014; Sommo & Chaskes 2013).

Findings from our PPI suggested that participants experienced high levels of poverty. This reinforces other data suggesting that people living with disabilities are among the most economically disadvantaged internationally (see Ofuani 2011) and the presence of a mutual relationship between poverty and disability (e.g. Banks & Polack 2014; Groce et al. 2011; Mitra et al. 2013). Most of our participants were involved in agriculture for livelihoods and ‘survival’, thus highlighting how agriculture has the potential to help alleviate poverty experienced by people living with and without disabilities, living in rural communities (Aranda-Jan 2021; NBS 2012). Our findings also corroborate other findings (e.g. Ogunlela & Mukhtar 2009; Ojiako et al. 2014) in the context of access to financial services, where most of our participants were not aware of such financial services that could assist their agricultural activities, as many of our participants relied on informal loans through families. Furthermore, participants reported that the type of agriculture and other related activities they could undertake without assistance was limited by their impairment. Moreover, as found in other research (e.g. Ojiako et al. 2014), the country would benefit from more focus on inclusive agricultural programmes, equipment and practices to empower and move individuals away from poor conditions by accessing opportunities and services guaranteed by law (i.e. the *Discrimination Against Persons with Disabilities (Prohibition) Act, 2018*), such as education and employment.

These findings emphasise the need for and importance of empowering people living with disabilities to be aware of their rights, empowering them with knowledge, practical employability and entrepreneurial skills and assistance, such as wheelchairs, walking sticks, basic spectacles, simple hearing aids and accessible agricultural equipment to become socially and economically independent (see Tsengu, Brodtkorb & Almnes 2000). Education and employment (including self-employment) play crucial roles for achieving positive economic outcomes for people living with disabilities. Economic participation in the form of employment (e.g. employment, supported employment and self-employment) not only improves financial independence of people living with disabilities but is also likely to improve their self-esteem and quality of life (Sango & Forrester-Jones 2017). This is especially because exclusion from economic and livelihood opportunities can negatively impact the psychosocial well-being and identity of those living with disabilities (Waddell & Burton 2006).

## Limitations

Limited data were collected that enabled potential differences that may be associated with urban or rural living to be explored. In addition, the perceived causation of disability did not explore in detail some important factors, such as genetic or accidental causation, which may guide the development of preventive policy. Our study included more male than female participants because more male farmers living with disabilities were found across the study location; therefore, future research in this area could benefit from purposefully including an equal number of female and male farmers.

## Conclusion and recommendations

This research explored the lived experiences of people living with disabilities involved in agricultural activities in northern Nigeria. It examined the types of opportunities available for farmers living with disabilities, including access to financial services, ownership of assets, decision-making, inclusive participation and social and cultural issues. The outcomes of this research are particularly important for strategy development for disability-inclusive agricultural and entrepreneurship programmes in northern Nigeria, such that people with disabilities are able to maximise economic empowerment opportunities that guarantee increased income and better livelihoods. To support this, the authors recommend the following:

- Increased partnership between agricultural development interventions with DPOs and rights-based organisations in the design and implementation of agricultural and entrepreneurship programmes targeting people with disabilities. Intervention activities should be designed in such a way that they are accessible to people living with disabilities. Part of such a strategy is outreach activities specifically targeting farmers living with disabilities and training of project-implementing staff on disability inclusion strategies.
- Increased synergy between agricultural development interventions and key private sector players to invest more in programmes supported with evidence-based research that function to intersect gender and disability in both conflict and post-conflict areas. Such synergies have huge potential for stimulating increased participation and employment opportunities, building skill sets and engendering entrepreneurship among people living with disability, ultimately improving their livelihoods.
- Agricultural development interventions can facilitate linkages between DPOs and farmers’ groups or cooperatives as a strategy for people living with disabilities to actively engage in farming and other related agribusinesses. In this way, farmers living with disabilities can better organise into producer groups through which they can acquire essential production inputs and receive support from various institutions, including banks and relevant institutions.
- Facilitate linkages between financial institutions with both farmers and people living with disabilities that engage in agricultural activities. This way, individuals can better maximise amongst the several agricultural financing schemes targeting smallholder farmers within their communities. This may include BOA, Bank of Industry (BOI), Central Bank of Nigeria (CBN) and relevant MFIs supporting agricultural and entrepreneurship financing within the communities.
- Disability-friendly tools and equipment will play a major role in terms of ease of wider and varied access and participation for farmers living with disabilities involved in agricultural activities.

## References

[CIT0001] Abang, T.B., 1988, ‘Disablement, disability and the Nigerian society’, *Disability, Handicap & Society* 3, 71–77. 10.1186/s40854-018-0112-2

[CIT0002] Abraham, T.W., 2018, ‘Estimating the effects of financial access on poor farmers in rural northern Nigeria’, *Financial Innovation* 4(1), 1–20. 10.1080/02674648866780061

[CIT0003] Acharya, S.S., 2006, ‘Sustainable agriculture and rural livelihoods’, *Agricultural Economics Research Review* 19(347-2016-16775), 205–218.

[CIT0004] Ahlenbäck, A., Lee., H. & Coe, S., 2020, *Disability inclusion helpdesk webinar: Mobile phone technology for disability-inclusive agricultural development, disability inclusion helpdesk research report No. 17*, Disability Inclusion Helpdesk, London.

[CIT0005] Aranda-Jan, C., 2021, *Inclusive digital agriculture: Making value chains work for farmers with disabilities*, viewed 07 March 2022, from https://www.gsma.com/mobilefordevelopment/resources/inclusive-digital-agriculture-making-value-chains-work-for-farmers-with-disabilities/.

[CIT0006] Audu, I.A., Idris, S.H., Olisah, V.O. & Sheikh, T.L, 2013, ‘Stigmatization of people with mental illness among inhabitants of a rural community in northern Nigeria’, *International Journal of Social Psychiatry* 59(1), 55–60. 10.1177/0020764011423180 22131198

[CIT0007] Banks, L.M. & Polack, S., 2014, *The economic costs of exclusion and gains of inclusion of people with disabilities*, International Centre for Evidence in Disability, London.

[CIT0008] Central Intelligence Agency, 2022, The world fact book, Central Intelligence Agency, Washington, DC, viewed 05 May 2016, from https://www.cia.gov/the-world-factbook/

[CIT0009] Cooper, S.A., Smiley, E., Morrison, J., Williamson, A. & Allan, L., 2007, ‘Mental ill-health in adults with intellectual disabilities: Prevalence and associated factors’, *British Journal of Psychiatry* 190, 27–35. 10.1192/bjp.bp.106.022483 17197653

[CIT0010] DFID, 2000, *Disability, poverty and development. Issue paper*, Department for International Development, London, viewed 08 April 2021, from http://www.dfid.gov.uk/pubs/files/disability.pdf/.

[CIT0011] Emerson, E. & Parish, S., 2010, ‘Intellectual disability and poverty: Introduction to the special section’, *Journal of Intellectual and Developmental Disabilities* 35, 221–223. 10.3109/13668250.2010.525869 21117879

[CIT0012] Etieyibo, E. & Omiegbe, O., 2016, ‘Religion, culture, and discrimination against persons with disabilities in Nigeria’, *African journal of disability* 5(1), 192. 10.4102/ajod.v5i1.192 28730043 PMC5433448

[CIT0013] Farnworth, C.R., Badstue, L., Williams, G.J., Tegbaru, A. & Gaya, H.I.M., 2020, ‘Unequal partners: Associations between power, agency and benefits among women and men maize farmers in Nigeria’, *Gender, Technology and Development* 24(3), 271–296.

[CIT0014] Grameen Foundation, 2014, *Poverty probability index*, viewed 07 May 2019, from https://www.povertyindex.org/free-tools.

[CIT0015] Groce, N., Kett, M., Lang, R. & Trani, J.F., 2011, ‘Disability and poverty: The need for a more nuanced understanding of implications for development policy and practice’, *Third World Quarterly* 32(8), 1493–1513. 10.1080/01436597.2011.604520

[CIT0016] Huyer, S., 2016, ‘Closing the gender gap in agriculture’, *Gender, Technology and Development* 20(2), 105–116. 10.1177/0971852416643872

[CIT0017] Imonikebe, B.U., 2010, ‘Constraints to rural women farmers’ involvement in food production in Nigeria’, *African Research Review* 4(3), 281–288. 10.4314/AFRREV.V4I3.60269

[CIT0018] Malli, M.A., Sams, L., Forrester-Jones, R., Murphy, G. & Henwood, M., 2018, ‘Austerity and the lives of people with learning disabilities: A thematic synthesis of current literature’, *Disability and Society* 33(9), 1412–1435. 10.1080/09687599.2018.1497950

[CIT0019] Mitra, S., Posarac, A. & Vick, B., 2013, ‘Disability and poverty in developing countries: A multidimensional study’, *World Development* 41, 1–18. 10.4324/9780429287657

[CIT0020] Montrose, 2017, ‘Persons with disabilities: Actors within agribusiness northern uganda transforming the economy through climate smart agri-business market development’, NU-TEC Report, Unpublished.

[CIT0021] Morgan, G.A., Barrett, K.C., Leech, N.L. & Gloeckner, G.W., 2019, *IBM SPSS for introductory statistics: Use and interpretation*, Routledge, New York, NY.

[CIT0022] Moyo, S., 2016, *Family farming in sub-Saharan Africa: Its contribution to agriculture, food security and rural development*, International Policy Centre for Inclusive Growth (IPC-IG), Brasilia.

[CIT0023] Murphy, N.A., Christian, B., Caplin, D.A. & Young, P.C., 2007, ‘The health of caregivers for children with disabilities: Caregiver perspectives’, *Child: Care, Health and Development* 33(2), 180–187. 10.4324/9780429287657 17291322

[CIT0024] National Bureau of Statistic (NBS), 2012, *Annual abstract of statistics*, National Bureau of Statistic, Abuja.

[CIT0025] Obiakor, F.E., 1990, ‘Special education policies in Nigeria: Cultural, socio-economic and political issues’, paper presented at the 68th Annual Convention of the Council for Exceptional Children, Toronto, 23–27th April, viewed 13 July 2019, from http://files.eric.ed.gov/fulltext/ED320375.pdf.

[CIT0026] Ofuani, A.I., 2011, ‘The right to economic empowerment of persons with disabilities in Nigeria: How enabled?’, *African Human Rights Law Journal* 11(2), 639–658.

[CIT0027] Ogunlade, I., Omotesho, K.F., Lawal, M.A. & Kehinde, F.B., 2016, ‘Determinants of level of participation of farmers in group activities in Kwara state, Nigeria’, *Gaziosmanpaşa Üniversitesi Ziraat Fakültesi Dergisi* 33(3), 21–27. 10.4324/9780429287657

[CIT0028] Ogunlela, Y.I. & Mukhtar, A.A., 2009, ‘Gender issues in agriculture and rural development in Nigeria: The role of women’, *Humanity and Social Sciences Journal* 4(1), 19–30.

[CIT0029] Ojiako, I.A., Idowu, A.O. & Ogbukwa, C., 2014, ‘Determinants of loan repayment behaviour of smallholder cooperative farmers in Yewa north local government area of Ogun state, Nigeria: An application of Tobit model’, *Journal of Economics and Sustainable Development* 5(16), 144–153.

[CIT0030] Oluwatoyese, O.P., Applanaidu, S.D. & Razak, N.A.A., 2016, ‘Macroeconomic factors and agricultural sector in Nigeria’, *Procedia-Social and Behavioral Sciences* 219, 562–570. 10.1016/j.sbspro.2016.05.035

[CIT0031] Sango, P.N., 2017, ‘Country profile: Intellectual and developmental disability in Nigeria’, *Tizard Learning Disability Review* 22, 87–93. 10.3109/13668250.2017.1310820

[CIT0032] Sango, P.N. & Forrester-Jones, R., 2017, ‘Spirituality and social networks of people with intellectual and developmental disability’, *Journal of Intellectual and Developmental Disability* 43(3), 1–11. 10.1108/TLDR-07-2016-0019

[CIT0033] Sango, T.J.D., 2013, ‘The role of traditional rulers in protracted communal conflicts in Nigeria’, Doctoral dissertation, University of Kent, Canterbury.

[CIT0034] Sheahan, M., Barrett, C.B. & Sheahan, M.B., 2014, ‘Understanding the agricultural input landscape in sub-Saharan Africa: Recent plot, household, and community-level evidence’, *World Bank Policy Research Working Paper* No. 7014, Washington, DC.

[CIT0035] Sommo, A. & Chaskes, J., 2013, ‘Intersectionality and the disability: Some conceptual and methodological challenges’, in *Disability and intersecting statuses*, S.N. Barnartt & B. Altman (eds.), vol. 7, pp. 47–59, Emerald Group Publishing Limited, Bingley.

[CIT0036] Swindale, A. & Bilinsky, P., 2006, *Household dietary diversity score (HDDS) for measurement of household food access: Indicator guide*, Food and Nutrition Technical Assistance Project, Academy for Educational Development, Washington, DC.

[CIT0037] Thomas, C., 2007, *Sociologies of disability and illness: Contested ideas in disability studies and medical sociology*, Palgrave Macmillan, Basingstoke.

[CIT0038] Toolbox, K., 2016, *Data collection tools for challenging environments*, viewed 09 July 2019, from https://www.kobotoolbox.org/.

[CIT0039] Trani, J.F. & Loeb, M., 2012, ‘Poverty and disability: A vicious circle? Evidence from Afghanistan and Zambia’, *Journal of International Development* 24, S19–S52.

[CIT0040] Tsengu, D.V., Brodtkorb, S. & Almnes, T., 2000, ‘CBR and economic empowerment of persons with disabilities’, in *CBR as part of community development: A poverty reduction strategy*, University College London, Centre for International Child Health, London, viewed 09 July 2019, from https://asksource.info/cbr-book/cbraspart_04.pdf.

[CIT0041] United Nations, 2006, ‘Convention on the Rights of Persons with Disabilities (CRPD)’, viewed 10 January 2022, from https://www.un.org/development/desa/disabilities/convention-on-the-rights-of-persons-with-disabilities.html 10.1515/9783110208856.203 18348362

[CIT0042] Waddell, G. & Burton, A.K., 2006, *Is work good for your health and well-being?* The Stationery Office, London.

[CIT0043] World Bank., 2009, *Gender in agriculture sourcebook*, World Bank, Washington, DC, viewed 07 March 2022, from https://documents1.worldbank.org/curated/en/799571468340869508/pdf/461620PUB0Box3101OFFICIAL0USE0ONLY1.pdf.

[CIT0044] World Health Organisation, n.d., *Disabilities*, viewed 05 January 2020, from https://www.who.int/topics/disabilities/en/.

[CIT0045] World Health Organization & World Bank, 2011, *World report on disability 2011*, World Health Organization, viewed 12 July 2019, from www.who.int/disabilities/world_report/2011/report.pdf.

